# Relationship between Contingent Negative Variation and afterimage duration in migraine patients

**DOI:** 10.3389/fneur.2024.1401212

**Published:** 2024-05-17

**Authors:** Simeon Giesen, Florian Rimmele, Tim P. Jürgens, Jörg Scheidt, Johannes Drescher, Ann-Kristin Leonhardt, Sophia Schulze, Birgit Harbeck, Wolfgang Meyer, Britta Müller, Peter Kropp, Armin Keller

**Affiliations:** ^1^Institute of Medical Psychology and Medical Sociology, University of Rostock Medical Center, Rostock, Germany; ^2^Department of Neurology, University of Rostock Medical Center, Rostock, Germany; ^3^Department of Neurology, KMG Hospital Güstrow, Güstrow, Germany; ^4^Institute for Informations Systems, University of Applied Sciences, Hof, Germany; ^5^III. Department of Medicine, University Medical Center Hamburg-Eppendorf, Hamburg, Germany; ^6^Amedes Experts, Endocrinology, Hamburg, Germany; ^7^Faculty of Medicine and Dentistry, Queen Mary University of London, London, United Kingdom

**Keywords:** migraine, after image, Contingent Negative Variation, pathomechanism, future migraine therapy, preemptive therapy

## Abstract

**Background:**

Abnormalities in electrocortical parameters and persistence of afterimage after visual stimulation are known to occur in migraine patients. The results of studies on Contingent Negative Variation (CNV) and afterimage persistence in migraine patients suggest a link between these two phenomena and a connection to the pathomechanism of migraine.

**Objectives:**

To date, no studies have investigated both afterimage duration and CNV parameters in the same subjects. The aim of this study was to investigate the relationship between the early component of CNV (iCNV) and the duration of the afterimage in migraine patients.

**Methods:**

Sixty seven migraine patients from the headache center of the University of Rostock Medical Center were examined for iCNV amplitude, iCNV habituation and afterimage duration. The subjects also completed questionnaires developed for this study and the MIDAS (Migraine Disability Assessment) questionnaire.

**Results:**

Associations were found between iCNV amplitude and afterimage duration and between habituation capacity and afterimage duration. A deficit in habituation capacity correlated with a significantly prolonged afterimage duration. Increased iCNV amplitude and prolonged afterimage duration were also significantly correlated.

**Conclusion:**

Conclusions about the pathophysiology of migraine can be drawn from the results of this study. The results support the hypothesis of cortical hyperexcitability as a consequence of a low pre-activation level, which may be a possible contributory cause of migraine. Furthermore, they allow assessment of whether the afterimage examination, which is easier and quicker to perform than the CNV examination, can be used as a diagnostic tool or as a parameter to monitor the course of therapy in people with migraine.

## 1 Introduction

Migraine is a disease with a high prevalence (1). A prevalence of 20% in women and 8% in men is assumed (2). The disease is associated with a high level of distress (3). The impairment caused by migraine is high, as are the restrictions on quality of life (4). Thus, migraine is one of the 20 most common causes of disabling worldwide (4). Comorbidities like major depression and panic disorder may accompany the disease (5).

The pathomechanism of migraine is still not fully understood (6, 7). Therefore, research into the pathomechanism and the effect of migraine medications plays an important role in improving future migraine therapy. Neuronal and vascular processes and the interplay between them lead to migraine (8–12).

Various methods allow conclusions to be drawn about cortical information processing. For example, various tests can be used to determine changes in cortical excitability in migraine patients. One example is sound-induced flash illusions, in which visual and auditory stimuli are presented simultaneously and cognitive processing can be evaluated (13).

## 2 Contingent Negative Variation and afterimage duration

### 2.1 Contingent Negative Variation

Migraine patients are known to have a higher amplitude of slow negative cortical potentials compared to healthy individuals. Slow negative cortical potentials are electrical potential shifts that can be measured in the summation of the simultaneous potential changes of individual neurons using an EEG. They are measured when the subject is expecting an external stimulus. This can be demonstrated by studies on the Contingent Negative Variation (CNV) (14–16). The Contingent Negative Variation, first described by Walter et al. in *Nature*, is an event-related, slow, negative cortical potential that occurs in individuals in an expectancy state. These potentials can be derived from an electrode positioned centrally on the scalp. It represents the planning of goal-directed behavior and is interpreted as attentional readiness (17). Furthermore, a reduced habituation effect on the early component of CNV, the so-called iCNV amplitude, is observed when a stimulus is repeatedly presented to people with migraine (15, 16). Regular monitoring of iCNV amplitude can help to predict future migraine attacks, which allows drug and non-drug interventions to be undertaken at an early stage (18–20). However, so far this prediction has been applied exclusively in studies because the determination of CNV parameters is technically very complex and time-consuming.

### 2.2 Afterimage duration

Besides the specificities regarding CNV components, migraine patients show a particularly long afterimage duration (12, 21, 22). Afterimage duration is characterized by the persistence of an afterimage induced by a visual stimulus. The origin of afterimage effect may lie at the cortical level (23, 24). While the majority of studies assume a prolonged afterimage effect in migraine patients, one study showed the opposite (25).

### 2.3 Links between CNV and afterimage duration

Both phenomena, *CNV amplitude* and *afterimage duration*, may provide insights into the pathomechanism of migraine. The hypothesis of a common etiological origin of the increased iCNV amplitude, the prolonged afterimage duration and the occurrence of headache can be derived from study results. People with migraine show a reduced cortical pre-activation level, which leads to a disturbance of the neuronal balance between inhibitory and excitatory processes and cortical information processing (26, 27). As a result, neuronal hyperexcitability occurs in the presence of non-painful sensory stimuli. The phenomena of habituation deficit and increased iCNV amplitude are due to a reduced level of pre-activation (26–31). Reduced cortical excitability requires greater synchronous activity of neurons to initiate the habituation mechanism. As a result, an habituation deficit and an increased iCNV amplitude are measured on average across all measurement series (26, 27, 32, 33). Cortical hyperexcitability may be a cause of prolonged afterimage in migraine patients (12). It leads to overactivation of the thalamus, which may be part of the pathogenesis of migraine (34). Visual phenomena in migraine patients can be explained by an overactivation of the *Corpus geniculatum laterale* as a component of the thalamus and the visual pathway.

### 2.4 Similarities in study results

Analogies between CNV amplitude and afterimage duration can be found. Kropp et al. demonstrated a decrease in iCNV amplitude with increasing age in subjects without migraine. This effect may be attributed to an increase in habituation with age, which can be interpreted as a sign of cerebral maturation. In people with migraine there was neither an increase in habituation nor a decrease in iCNV amplitude (33). There is evidence that increasing age in healthy subjects is associated with a reduction of afterimage duration. Again, this phenomenon is not observed in migraine patients. There are only small differences in afterimage latency between people with migraine and healthy young subjects, whereas the difference increases with age (12). As a result, the long afterimage duration in adult migraine patients might be an indication of altered cortical development and cerebral maturation—just like the development of iCNV amplitude.

Considering the migraine cycle, the two phenomena of iCNV amplitude and longer afterimage duration depend on the migraine interval. The level of iCNV amplitude varies in relation to the migraine interval, allowing changes in iCNV amplitude to be measured over the progression of a migraine cycle. Increased iCNV amplitudes are measured during the interictal interval and especially one day before a migraine attack, while after a migraine attack these amplitudes are equal to or lower than those of healthy subjects (18, 19, 32). Luedtke et al. observed a periodic development in the duration of afterimages. Longer afterimage durations were measured in preictal migraine patients compared to interictal migraine patients (25). However, it should be noted that key findings of this study contradict those of other studies. In contrast to other studies on afterimage duration or other visual phenomena in migraine patients (12, 21), Luedtke et al. found a less-pronounced afterimage duration in people with migraine compared to healthy subjects. The authors concluded a periodic change in the balance of cortical neuronal excitation and inhibition during migraine cycles (25).

All these results were obtained in studies investigating either CNV parameter expression or afterimage duration in migraine patients. There is also a lack of studies investigating these phenomena in the same subjects.

## 3 Methods

### 3.1 Sample

Subjects were recruited from the headache center of the University of Rostock Medical Center. The study was approved by the ethics committee of the University of Rostock Medical Center (registration numbers: A 2011–0029 and A 2017–0187). Sixty seven patients with migraine with and without aura (maximum of 14 migraine days/month, disease duration at least one year, at least five migraine attacks prior to study participation) were examined. Diagnosis was made according to the third edition of the *International Classification of Headache Disorders (ICHD-3)*. Exclusion criteria were mental illness, pregnancy and medication overuse. Sample size requirements were derived from sample sizes in various studies of CNV and afterimage duration in people with migraine (18, 35).

### 3.2 Study design

**Figure 1:**
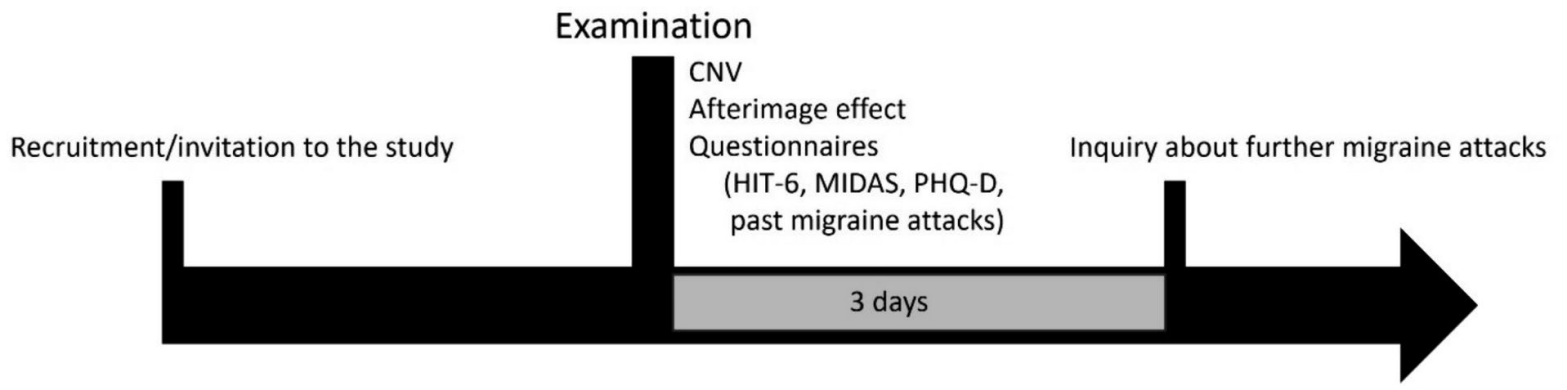
Study design.

Contingent Negative Variation and afterimage duration were measured in each participant (Figure 1). In addition, participants completed a questionnaire designed for this study, including the number of migraine days, time since last migraine attack (in days), triggers and duration of the disease (in months). Migraine occurrence in the two-day period before and after the study was assessed using a pain diary, the questionnaire and telephone interviews. We only used data from people who had no migraine attack for at least two days before or after the study, because the amplitude of iCNV can be influenced by a migraine attack (18, 19).

In addition, participants completed two other questionnaires. The HIT-6 (*Headache Impact Test*) is a questionnaire designed to assess headache-related impairment in migraine patients (36) and measures the negative impact of headaches on daily life (37). The test examines how people subjectively rate their well-being or pain, their ability to concentrate and any limitations in their daily activities (see questions on the HIT-6). A higher score indicates a greater degree of impairment due to pain. Scores can range from a minimum of 36 to a maximum of 78. Headache-related disability can also be assessed using the MIDAS (*Migraine Disability Assessment*), which consists of a scale ranging from grade I (little or no disability) up to five days of migraine to grade IV (severe disability) up to 21 or more days of migraine. The last three months are assessed (see MIDAS questions). While the HIT-6 score is mainly influenced by pain intensity, the MIDAS score is mainly influenced by the number of headache days (38).

### 3.3 Acquisition of CNV

CNV parameters were measured according to a previously developed protocol to ensure uniform test conditions (light, room atmosphere). The 10–20 system published by Herbert Jasper in Science in 1935 (39, 40) was used to determine the electrode positions. Before applying the electrodes, the skin sites were disinfected and coated with an electrically conductive paste supplied by NIHON KOHDEN. Ring electrodes coated with silver-silver chloride (Ag/AgCl) were used as electrodes. Six electrodes were used for each measurement: the area over the Cz (*Central zero*), two areas over the forehead (Fp1 and Fp2), two electrodes on the *Processūs mastoidei* and one electrode for recording an electro-oculogram (EOG). Position Cz was used for data analysis. The two linked mastoid electrodes served as reference electrodes. The electrode on the forehead served as ground electrode. The remaining electrodes were used to detect artifacts of the *Musculus orbicularis oculi* or movements of the eyeballs.

The CNV measurement consisted of 32 Go and 8 NoGo trials. The Go trials consisted of an acoustic stimulus S1 (warning stimulus) with a frequency of 1,000 Hz (medium-high pitch) and a duration of 100 ms. After 3 s, S1 was followed by the presentation of a sound S2 (imperative stimulus) with a frequency of 2,500 Hz (high pitched tone) and a duration of up to 2,500 ms. The subject had to respond to this second stimulus as quickly as possible by pressing a button, after which S2 was switched off. A *NoGo section*, to which the subject was not required to respond, consisted of an acoustic stimulus at a frequency of 200 Hz (low frequency tone). This tone was not followed by an imperative stimulus. The presentation of the *Go* and *NoGo stimuli* was controlled by the *E-Prime v2.0* program (E-Prime, 2002) in randomized order. The CNV was derived using an EEG amplifier from *Brain Products*. The *Brain Vision Recorder* (version 1.20.0601) from *Brain Products* was used to record the data.

The measured electrocortical data were analyzed using the *Brain Vision Analyzer* (version 2.1.1.327) software from *Brain Products*. A high-pass filter (0.03281 Hz, slope unit: 12 dB/octave), a low-pass filter (30 Hz, slope unit: 12 dB/octave) and a band-stop filter (50 Hz) were used to process the raw EEG data. During segmentation, 32 sequences of equal length were formed. The baseline correction was followed by a qualitative artifact correction in which sequences with high artifact content were removed after reviewing the 32 sequences. The number of rejected trials was recorded.

The iCNV amplitude was determined using the procedure described by Böcker et al. For this purpose, the respective amplitude maximum was determined in the time interval between 550 and 750 ms after stimulus S1 presentation. The time of this maximum amplitude represents the center of a constructed time window that also included a time period of 100 ms before and after the maximum. The mean amplitude during this time interval was defined as the iCNV amplitude (33, 41).

To determine the habitability coefficient, 32 trials were assigned to eight blocks of four trials each. The amplitude levels in each block were averaged. If there were fewer than 32 trials left due to artifacts, eight blocks with fewer than four trials each were formed, taking care to ensure a homogeneous distribution of trials in the blocks. Finally, the habituation coefficient was determined by regression analysis. It was described by a linear equation of the form *y = ax + b* (a = slope parameter, b = intercept of the y-axis). Parameter a represents the slope parameter, which is closely related to the habituation coefficient, while parameter *b* describes the point of intersection with the y-axis. This approach corresponds to the procedure used in several studies (32, 33).

### 3.4 Acquisition of afterimage

**Figure 2:**
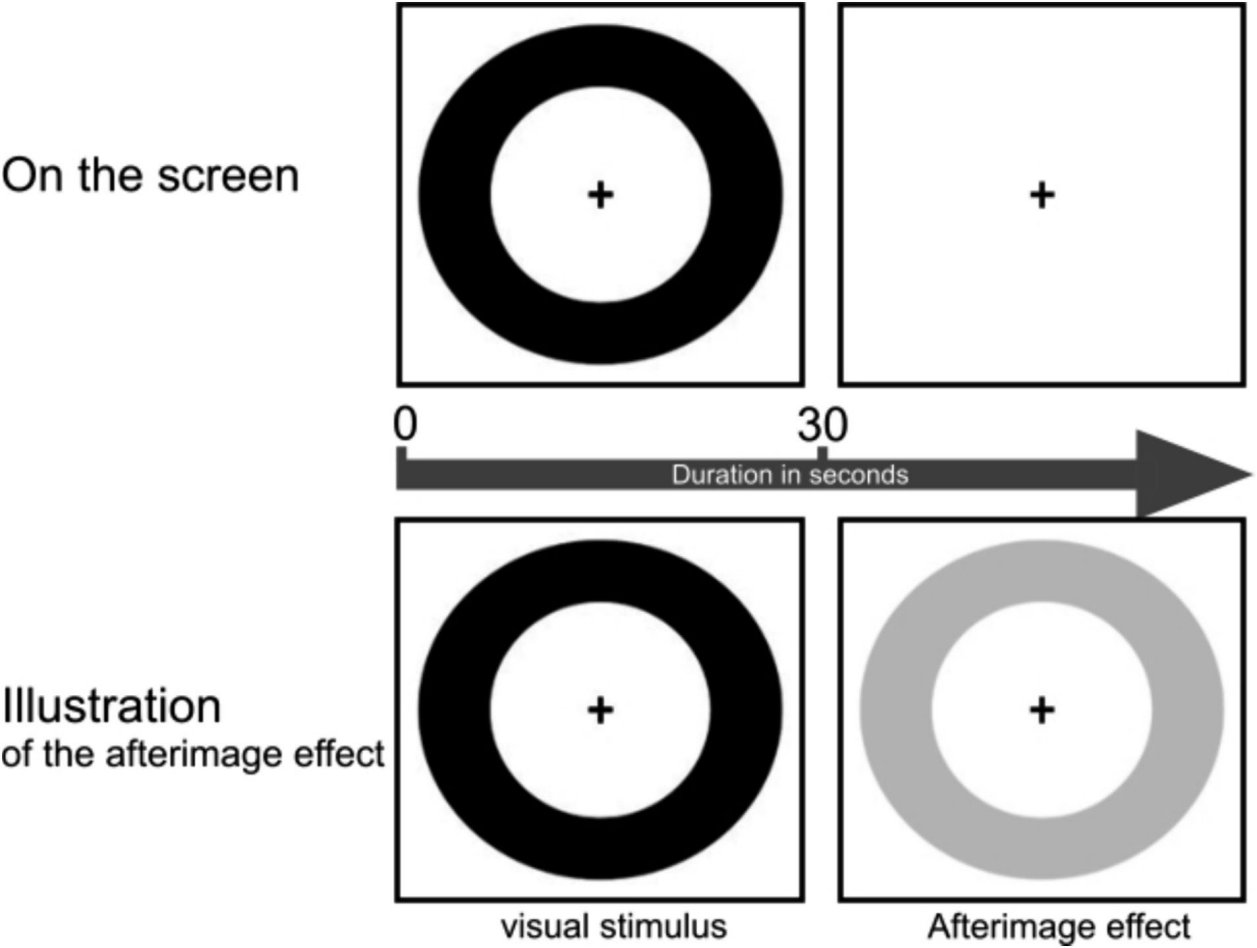
Procedure of the afterimage duration test. The upper part of the figure shows the 30-s presentation of the visual stimulus. The lower part illustrates the appearance of the afterimage in the subject’s perception after the visual stimulus disappeared.

The afterimage duration was determined - similar to other studies (21, 22, 35) — using an *iPad Pro* (Model A1584) with a display size of 12.9 inches (32.78 cm screen diagonal) positioned 60 cm away from the subject’s eyes. A black circular ring with a cross in the center was presented to each patient for a period of 30 s. After this period, the subject perceived an afterimage (Figure 2). The subject was asked to report the time at which the afterimage disappeared completely. It should be noted that there was no eye blinking during this time, as this can affect the duration of the afterimage (42). The duration of the afterimage was measured in seconds.

The expression of afterimage duration was determined according to a protocol established for this study. This allowed comparable conditions between subjects and between the first and second examination of each subject. A room with a quiet atmosphere and uniform lighting conditions was used for the study. The subjects sat on an examination chair in a relaxed position. All subjects were asked if they had any visual impairment that would have prevented them from adequately perceiving the visual stimulus.

### 3.5 Statistical analysis

*SPSS* (Version 27) was used for the statistical analysis of the data. Data were analyzed for all subjects not having migraine attacks 2 days before and after the examination. This was determined by using questionnaires and telephone calls. All data used therefore originate from participants who were in the interictal interval. Parametric tests were used to test for normal distribution using the *Kolmogorov–Smirnov* and *Shapiro–Wilk* tests. In addition, the homogeneity of the distribution of demographic and disease-related characteristics in each sample was tested.

We divided the subjects into the two groups habituation/sensitization or long (> 6 s)/short (< 6 s) afterimage effect. Hypotheses were tested using ANOVA. As the hypotheses were directional, one-tailed tests were always performed and a probability of error of <5% was assumed.

## 4 Results

### 4.1 Distribution of demographic and disease-related characteristics

Table 1 presents demographic and disease-related characteristics in subjects with differences in habituation expression. There are no significant differences between the two groups. Among the subjects with habituation, 37.5% had migraine with aura. Among the subjects with dishabituation, 42.9% had a migraine with aura. There is no significant difference in habituation between migraine patients with and without aura (Table 1).

**Table 1:** Comparison of demographic and disease-related characteristics between subjects with habituation versus dishabituation.

	Subjects with habituation (*n* = 11)	Subjects with dishabituation (*n* = 17)	Significance *p*
Age (years)	51.25	50.36	0.877 (n.s.)
Migraine days per month	7.31	7.21	0.952 (n.s.)
Pain intensity (1–10)	7.13	7.04	0.899 (n.s.)
Duration of illness (months)	162.57	219.00	0.375 (n.s.)
HIT-6	62.63	63.21	0.845 (n.s.)
MIDAS (score)	61.64	67.38	0.855 (n.s.)

N, Number; HIT-6, Headache Impact Test; MIDAS, Migraine Disability Assessment; n.s., not significant (*t*-test; *p* > 0.05).

Table 2 illustrates demographic and disease-related characteristics of subjects with different degrees of afterimage duration. There are no significant differences in these characteristics between subjects with high and low afterimage duration. Among the subjects with long afterimage duration, 55.0% had migraine with aura. Among the subjects with short afterimage duration, 38.5% had a migraine with aura. There is no significant difference in afterimage duration between migraine patients with and without aura (Table 2).

**Table 2:** Comparison of the demographic and disease-related characteristics between the groups with strong versus less pronounced afterimage duration.

	Subjects with long afterimage duration (*n* = 11)	Subjects with short afterimage duration (*n* = 18)	Significance *p*
Age (years)	47.37	55.54	0.133 (n.s.)
Migraine days per month	7.75	7.54	0.894 (n.s.)
Pain intensity (1–10)	6.94	7.31	0.577 (n.s.)
Duration of illness (months)	215.50	166.15	0.449 (n.s.)
HIT-6	66.63	62.08	0.109 (n.s.)
MIDAS (score)	43.23	97.75	0.083 (n.s.)

n, Number; HIT-6, Headache Impact Test; MIDAS, Migraine Disability Assessment; n.s., not significant (*t*-test; *p* > 0.05).

Prophylactic agents were used by 14 subjects. Six patients used beta-blockers, five patients used amitriptyline, and three patients used topiramate.

### 4.2 Drop-out

**Figure 3:**
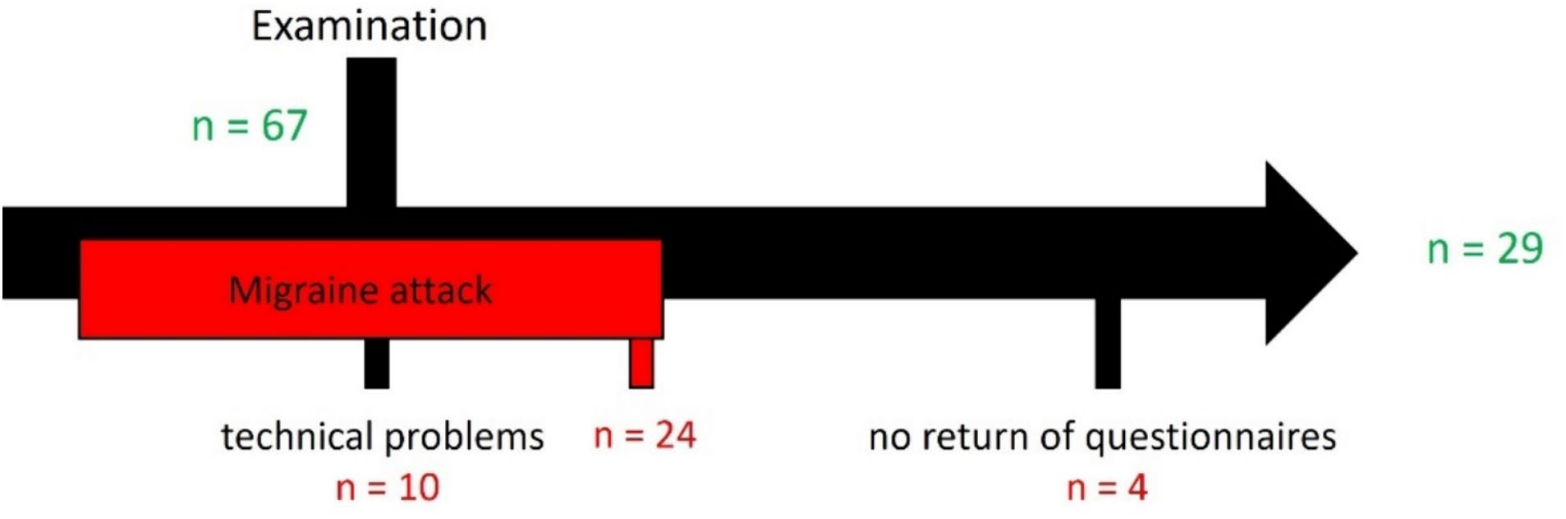
Drop-out.

Data from 29 of the 67 subjects were used for further analysis (Figure 3). The afterimage duration and iCNV amplitude results of 24 subjects could not be included because the subjects reported a migraine attack within two days before or after the study day. A migraine attack can affect the level of iCNV amplitude and the habituation coefficient (18, 19, 32, 43) as well as the afterimage duration (25). Technical problems occurred in ten subjects. Four subjects did not return their questionnaires. No subject reported insufficient visual acuity, which would have prevented evaluation of the afterimage duration.

### 4.3 Relation between habituation capacity and afterimage duration

To investigate the relationship between afterimage duration characteristics and habituation capacity in migraine patients, subjects were divided into two groups, habituated (habituation coefficient < 0) and dishabituated (habituation coefficient > 0), based on their iCNV amplitude-related habituation capacity.

**Figure 4:**
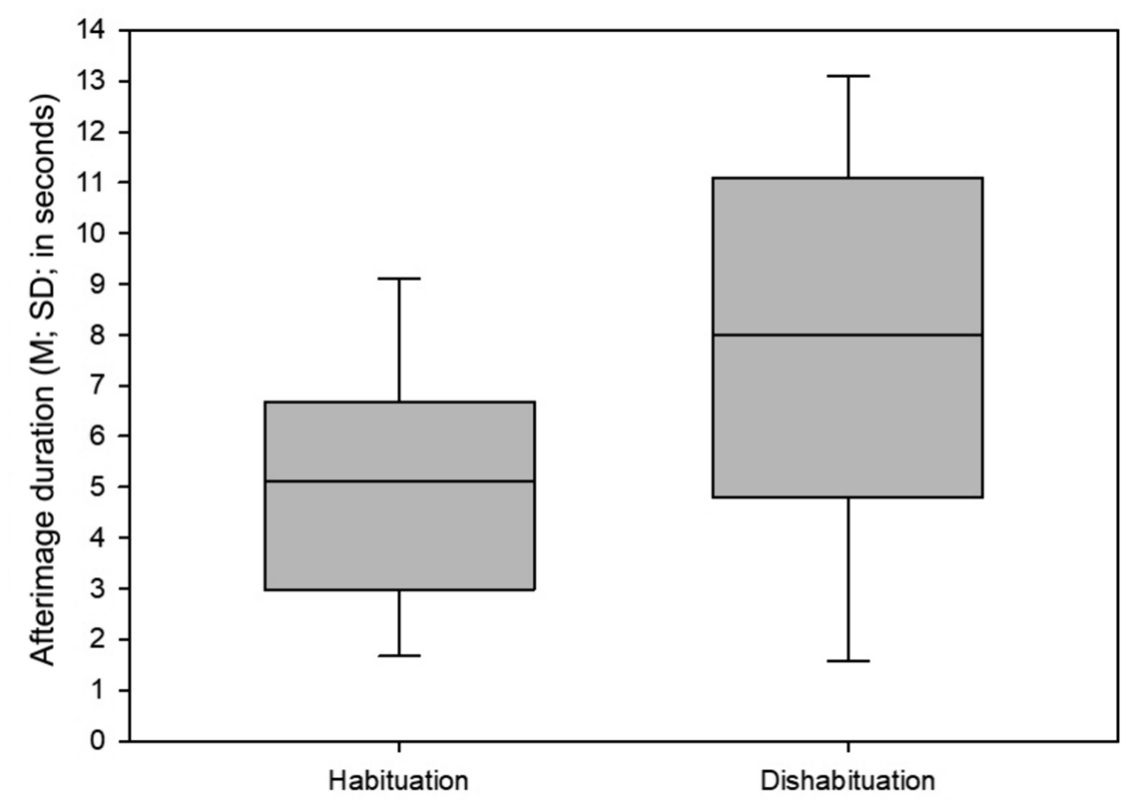
Mean and standard deviation of afterimage duration time in migraine patients with habituation and dishabituation. M, Mean; SD, standard deviation.

Significant differences (*p* = 0.013) in the duration of the afterimage evoked by a black visual stimulus (black circular ring) were detected between subjects with identified habituation and sensitization regarding the iCNV amplitude (Table 3). Afterimages were reported to last for a shorter time in subjects with pronounced habituation (M_afterimage_ = 5.11 s; SD_afterimage_ = 2.36 s) compared to subjects with sensitization (M_afterimage_ = 8.17 s; SD_afterimage_ = 3.86 s) (Figure 4).

**Table 3:** Afterimage duration in subjects with habituation versus dishabituation.

	Habituation capacity	N	M	SD	MD	*p*
Afterimage duration (in seconds)	Dishabituation	17	8.17	3.86	3.06	0.013*
	Habituation	11	5.11	2.36		

N, Number of patients; M, mean; SD, standard deviation; MD, Mean difference; *p*, *p*-value. *(*p* < 0.05).

As there are only a few studies on the afterimage effect to date and the experimental setup differs, no direct comparisons can be made between the results of different studies.

### 4.4 Relation between iCNV amplitude and afterimage duration

Amplitudes of the early components of the CNV are compared between subjects with long (≥6 s) and short (<6 s) afterimage duration. ANOVA revealed a significant difference (*p* = 0.011) between the two groups in the amplitude of iCNV (Table 4).

**Table 4:** iCNV amplitude in relation to afterimage duration.

	Afterimage duration	N	M	SD	MD	*p*
iCNV-Amplitude (in μV)	Long	18	−11.59	3.17	−2.54	0.011*
	Short	11	−9.05	1.79		

N, Number of patients; M, mean; SD, standard deviation; MD, Mean difference; *p*, *p*-value. *(*p* < 0.05).

**Figure 5:**
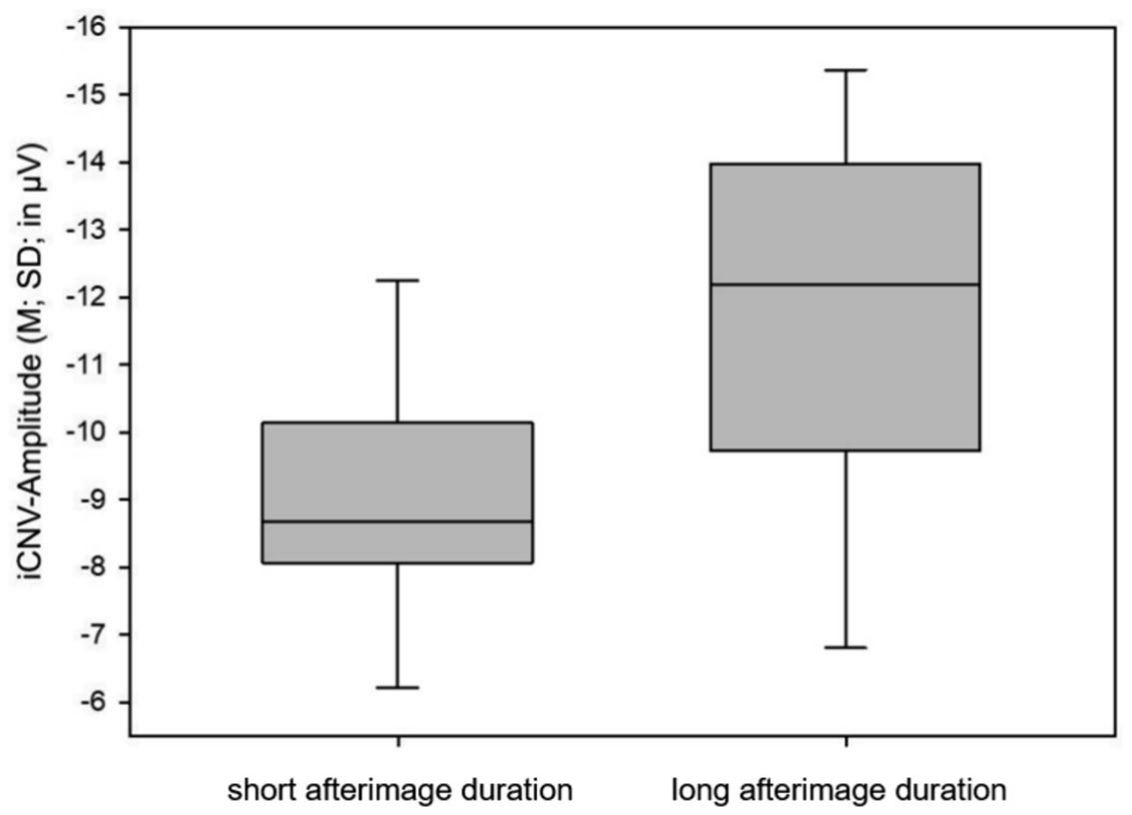
Mean and standard deviation of iCNV amplitude in migraine patients with long and short afterimage duration. M, mean; SD, standard deviation.

Subjects experiencing long afterimage duration showed significantly higher iCNV amplitudes (M_iCNV_ = −11.59 μV; SD_iCNV_ = 3.17 μV) than subjects reporting an afterimage persisting for less than 6 s (M_iCNV_ = −9.05 μV; SD_iCNV_ = 1.79 μV) (Figure 5). The iCNV values determined in migraine patients are consistent with previous studies. In healthy volunteers, iCNV values of around −4.8 μV have been measured in previous studies (14).

## 5 Discussion

The results confirm the hypothesis of a correlation between the two examined parameters iCNV amplitude and afterimage duration. Long afterimage duration is associated with a high (more negative) iCNV amplitude. This may be related to the pathophysiology of migraine. In addition, the results allow a model of the pathophysiology of migraine to be constructed.

### 5.1 Model of the pathophysiology of migraine

**Figure 6:**
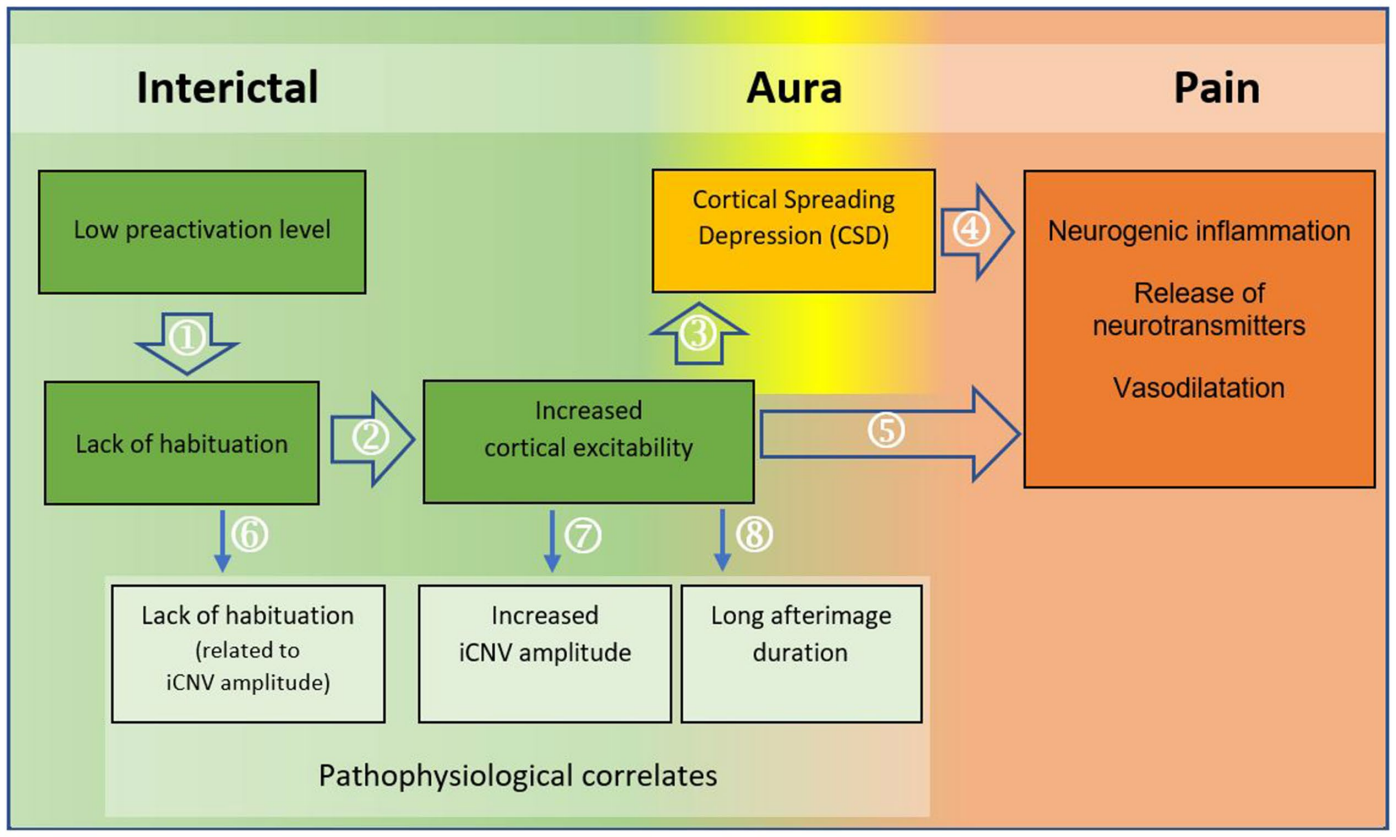
Model of the pathomechanism of migraine with regard to the migraine phases. The flowchart illustrates a model of the pathomechanism of migraine. Arrows 1–5 represent the pathomechanism; arrows 6–8 show the pathophysiological origins of the (electrophysiologically) measurable findings in migraine patients. Sources (with attribution to the components in the model): ① (26, 27); ② (44); (32, 33); ③ (8, 45); ④ (11, 46–55); ⑤ (9); ⑥ (27, 33); ⑦ (33); ⑧ (12).

The results of several studies can be used to construct a model of the pathomechanism of migraine, supported by the associated pathophysiological correlates (Figure 6). People with migraine have a lower level of baseline neuronal excitation than healthy people (26). This low level of pre-activation allows a greater range of suprathreshold activation before reaching the threshold of inhibitory processes. As a result, the low baseline cortical excitation requires greater synchronous neuronal activity to achieve the onset of the habituation mechanism (26, 27). The habituation deficit in migraine patients demonstrated in numerous studies (15, 16, 56) may thus be related to the low cortical pre-activation level (26, 27). Habituation is a natural mechanism that is responsible for a decrease in the intensity of the post-stimulus orienting response when an identical stimulus is repeatedly perceived with persistently the same intensity (44). Critically, the habituation process protects the brain from too much information and too much cortical activation. Therefore, reduced habituation capacity and impaired selective information processing lead to increased excitability of cortical neurons (32, 33, 44). Another possible explanation for increased cortical excitability is an insufficiency of neuronal mitochondria, which leads to an energy deficit and an insufficient capacity for habituation (1, 12, 21, 57–59).

Increased cortical neuronal excitability may be responsible for the development of *Cortical Spreading Depression* (CSD). It has been suggested that the high number of de-and repolarizations leads to the accumulation of extracellular potassium, which promotes the development of CSD (8, 45). Although CSD occurs concurrently with the aura phase, it is thought to underlie not only the aura symptomatology but also the delayed headache phase of migraine (54). Thus, the increased trigeminal nociception caused by vasodilatation and neurogenic inflammation can be understood as a result of *Cortical Spreading Depression* (11, 12, 46–49, 51–55).

Consequently, as shown in the flowchart below (Figure 6), imbalances at the neuronal level lead to changes at the vascular level. It is likely that the link between neuronal and vascular processes plays a crucial role in the pathomechanism of migraine (9).

The origins of the pathophysiological correlates can be localized using the model. The cortical habituation deficit and increased neuronal excitability are manifested in the habituation deficit related to iCNV amplitude (27, 33) and the increased amplitude of the early CNV component (33). It is reasonable to assume that the increased iCNV amplitude in people with migraine is — at least in part — due to the habituation deficit. The early amplitude of the CNV does not decrease sufficiently during repetitions of the same stimulus. As a consequence of the habituation deficit, the amplitude of iCNV averaged over all trials is increased in migraine patients compared to healthy subjects (32, 33).

The long afterimage duration in migraine patients can be attributed to increased cortical excitability with reduced baseline activation (12). It appears that reduced habituation capacity is not only the cause of a migraine attack, but also of the increased amplitude of iCNV, the habituation deficit associated with iCNV amplitude and the prolonged afterimage latency.

The associations demonstrated in this study are consistent with the results of studies providing evidence for an origin of migraine in an altered pre-activation level (26, 27) or altered neuronal homeostasis caused by a neuronal mitochondrial deficit (1, 12, 21, 57–59). Furthermore, these findings support the hypothesis that the altered CNV parameters (27, 32, 33) and the very long afterimage duration (12, 60) in migraine patients are related to the pathophysiology of migraine. As this study has demonstrated a relationship between afterimage duration and CNV parameters, it is reasonable to conclude that both phenomena may play a crucial role in the study and treatment of migraine.

Consequently, it is reasonable to assume that periodic changes parallel to migraine phases can be detected not only by looking at iCNV amplitude, but also by examining the degree of afterimage duration. The identification of significant correlations between habituation coefficient and afterimage duration, or iCNV amplitude and afterimage duration, suggests parallel periodic changes in these phenomena during a migraine interval.

Previous studies have documented separate observations only for the periodicity of CNV amplitude (18, 19, 32, 43) *or* afterimage duration (25). Furthermore, as mentioned above, there are limitations to the interpretation of Luedtke et al.’s study on the periodicity of afterimage duration in migraine patients. Therefore, by considering the relationship between CNV parameters and afterimage duration, this study provides complementary findings to previously-published study results.

### 5.2 Improvement of migraine therapy

#### 5.2.1 Theory of migraine pathomechanism

An association between afterimage duration and CNV parameters supports the hypothesis of a similar or common origin of increased iCNV amplitude, habituation deficit and highly pronounced afterimage duration in people with migraine. These phenomena can be attributed to increased cortical pre-activation and cortical hyperexcitability. The theory of increased cortical excitability is supported by the results of this study.

#### 5.2.2 Therapy progress monitoring

It is already known that pharmacological therapy can influence the parameters of CNV (61–64). However, CNV assessment is not used as a diagnostic tool or for monitoring disease progression. This is partly due to the time- and personnel-intensive nature of the examination. As afterimage duration correlates with iCNV amplitude and habituation, it can be discussed as an examination tool that can be used for therapy monitoring.

#### 5.2.3 Early detection of a future migraine attack

The predictive value of iCNV amplitude has already been demonstrated in studies, as it can be used to predict an upcoming attack. This knowledge could be used therapeutically, for example, to prevent the development of migraine through early use of relaxation exercises (18, 19). Taking analgesics early, before a migraine attack, may also improve their effectiveness (20). From the demonstrated relationship between iCNV amplitude and afterimage duration, we can conclude that afterimage duration could be used to predict a future migraine attack.

Determination of afterimage duration has several advantages compared to CNV examination: It is less extensive, personnel-and time-intensive than the determination of CNV components and does not require extensive technical equipment. Therefore, it is conceivable that the determination of the afterimage duration could be performed independently by the patient at home after sufficient training. In addition, the simplicity of the method allows more frequent and shorter measurement intervals, which can improve the quality of the prediction. An app developed for people with migraine could predict the onset of an upcoming migraine attack (before prodromes occur) by analyzing the afterimage duration, allowing the patient to take a drug or non-drug prophylactic therapy. It may also enable the patient to identify further prodromes, since the time of occurrence of the prodromes can be determined in a comprehensible way by analyzing the afterimage duration.

Finally, as a result of more efficient treatment, medication overuse due to ineffective drug therapy, which can lead to the chronicity of migraine (65), can be prevented more often and more effectively. Effective treatment could reduce the direct and indirect costs of the disorder. Moreover, effective treatment can improve the quality of life of migraine patients and reduce their disabilities.

### 5.3 Limitations

The subjects do not represent the average characteristics of the overall population of people with migraine. Patients from the headache center at the University of Rostock Medical Center who were included in this study generally have a disease history of several years, a high number of migraine days and a long duration of a migraine attack. In addition, most patients had been pre-treated with medication. This may have had an effect on CNV parameters (61–64).

Further limitations arise from the examination methods and conditions used in this study, as well as the study design. Considering the periodicity of iCNV parameters (18–20) and the afterimage duration (22), the measured values are influenced by both the time of day and the time elapsed since the last attack. We minimized this influence by excluding from the analysis the data of all subjects who had experienced a migraine attack within two days prior to the study.

A separate analysis of the afterimage duration of migraine patients with and without aura was not performed. The existence of these differences can be explained by the fact that the pathomechanisms are likely to be different between migraine patients with and without aura (12, 21, 22). Therefore, we cannot exclude the possibility that a study with a larger number of subjects in each group might find differences in afterimage duration or the relationship between it and CNV parameters.

Our study is the first study to investigate the relationship between CNV and the afterimage effect in migraine patients. Due to the exploratory nature of the study, we did not analyze migraine patients with episodic and chronic migraine separately. However, as there may be differences between these two groups, patients with episodic and chronic migraine should be considered separately in the future.

The determination of the afterimage duration is more prone to error than objective measurement methods, as the active cooperation and concentration of the subject is essential. It is therefore likely that the patient’s reaction time has an influence on the measured afterimage duration. In addition, afterimage determination can be influenced by movements of the inner eye muscles and blinking (42).

## 6 Conclusion

The prediction of migraine attacks will play a decisive role in future therapy. Our results show that the examination of the afterimage can be suitable for this. Effective prediction enables patients to live a more symptom-free life and prevents excessive use of medication.

The influence of migraine phase, course, and treatment on the duration of afterimages has not yet been studied in detail. Therefore, we suggest that future studies should further investigate the characteristics of afterimage duration in people with migraine. In particular, the prognostic value of the afterimage duration should be investigated using a daily measurement, if possible, to reduce the influence of measurement errors related to the afterimage duration (42).

## Data availability statement

The raw data supporting the conclusions of this article will be made available by the authors, without undue reservation.

## Ethics statement

The studies involving humans were approved by ethics committee of the University of Rostock Medical Center. The studies were conducted in accordance with the local legislation and institutional requirements. The participants provided their written informed consent to participate in this study.

## Author contributions

SG: Writing – original draft. FR: Writing – review & editing. TJ: Writing – review & editing. JS: Software, Writing – review & editing. JD: Software, Writing – review & editing. A-KL: Writing – review & editing. SS: Writing – review & editing. BH: Writing – review & editing. WM: Writing – review & editing. BM: Formal analysis, Writing – review & editing. PK: Writing – review & editing. AK: Writing – review & editing.
